# Thermal Response of Jointed Rock Masses Inferred from Infrared Thermographic Surveying (Acuto Test-Site, Italy)

**DOI:** 10.3390/s18072221

**Published:** 2018-07-10

**Authors:** Matteo Fiorucci, Gian Marco Marmoni, Salvatore Martino, Paolo Mazzanti

**Affiliations:** 1 Earth Sciences Department of “Sapienza”, University of Rome and CERI—Research Centre for Geological Risks, P.le Aldo Moro n.5, I-00185 Rome, Italy; salvatore.martino@uniroma1.it (S.M.); paolo.mazzanti@uniroma1.it (P.M.); 2 NHAZCA S.r.L., Spin-off Company of “Sapienza” University of Rome, Via Vittorio Bachelet n.12, I-00185 Rome, Italy

**Keywords:** IR-thermography, rock masses, slope stability, thermal behaviour, thermomechanics

## Abstract

The Mediterranean region is affected by considerable daily and seasonal temperature variations due to intense solar radiation. In mid-seasons, thermal excursions can exceed tens of degrees thus influencing the long-term behaviour of jointed rock masses acting as a preparatory factor for rock slope instabilities. In order to evaluate the thermal response of a densely jointed rock-block, monitoring has been in operation since 2016 by direct and remote sensing techniques in an abandoned quarry in Acuto (central Italy). Monthly InfraRed Thermographic (IRT) surveys were carried out on its exposed faces and along sections of interest across monitored main joints. The results highlight the daily and seasonal cyclical behaviour, constraining amplitudes and rates of heating and cooling phases. The temperature time-series revealed the effect of sun radiation and exposure on thermal response of the rock-block, which mainly depends on the seasonal conditions. The influence of opened joints in the heat propagation is revealed by the differential heating experienced across it, which was verified under 1D and 2D analysis. IRT has proved to be a valid monitoring technique in supporting traditional approaches, for the definition of the surficial temperature distribution on rock masses or stone building materials.

## 1. Introduction

Small-scale landslides as falls, slides or topplings are the most common mechanism involving jointed rock masses in correspondence with cliff or steep-slopes [1,2,3,4]. These slopes can be kinematically predisposed to instabilities because of their pervasive jointing conditions and orientation, which controls both the strength and stiffness of the discontinuous medium, as well as the sizing of mobilised blocks. Based on these predisposing conditions, short-term transient phenomena, such as heavy rainfalls [5] or dynamic inputs (i.e., earthquakes or anthropic vibrations), can represent triggers able to lead the slope to failures, acting mutually on resistances and disturbing forces [6,7,8,9,10]. In addition to these common impulsive triggers [11], under specific climatic conditions where consistent thermal excursion exists, rock masses can also react to continuous cyclical thermal inputs, as the one due to solar radiation, which can operate on wider time-windows configuring as a preparatory factor for rock block failure [12].

Most of the InfraRed Thermographic (IRT) applications in Earth Sciences concern the monitoring of volcanic eruption [13] and of volcanic systems [14,15], the quantification of magma effusion rates [16,17,18] or the detection of temperature anomalies related to presence of geothermal fields or fluid migration [19,20,21] as well as of fumarolic emissions [22,23], even though the more widespread application of the IRT deals with building maintenance and monitoring for preservation of civil engineering infrastructures [24,25,26]. Despite the diffuse application of IRT monitoring, its application to the study of rock masses and related slope instability processes is still limited [27,28,29]. Pioneering application of this technique was tested to the study of the integrity of concretes or rock masses [30,31]. Such studies laid the basis for the assessment of jointing conditions [32] and rock mass geotechnical characterisation [28], aimed to the description of the proneness of rock slopes to failure [33,34,35,36] or to assess the geological hazard due to the presence of fast landslides [37].

Thermal forcing which continuously acts on jointed rock masses can induce permanent deformations. Thermo-mechanical effects are indeed referred in literature to the results of cycles of expansion and contraction in the rock matrix, which can operate over long time, inducing cumulative stress concentration along joints and promoting irreversible strains [38]. Cyclical thermal stresses are regarded to operate as microstructural fatigue processes [39,40,41] responsible for mechanical weathering of the rock interface able to induce plastic strain [42]. These effects are not limited to the shallowest portions of the rock masses but they can extend more deeply below the thermal active layer [43,44] as the result of propagation of existing cracks at its tip by thermally-induced stresses [45]. Several authors already investigated the possible causes of cumulative thermo-mechanical induced displacement along joints by means of wedging-driven mechanism [46,47,48]. Despite the suitability of the model for specific case studies, the occurrence of thermo-mechanical induced strains is not always limited to the presence of a driving wedge able to push rock-blocks over planar rough surfaces.

The comprehension of thermo-mechanical effects in jointed rock masses requires the definition of both the amplitude of thermal action and distribution of near-surface temperatures [49], which are mostly controlled by climatic effects (i.e., daily and seasonal thermal variations), jointing conditions, as well as it exposure to thermal perturbations [44]. It was demonstrated that across discontinuous jointed media, primary porosity and crack density can influence the heat propagation within a rock mass and particularly affecting the surficial cooling rate at its interface [34,50]. Natural diurnal or annual temperature oscillations can induce long-term thermo-mechanical effects on rock masses able to influence the integrity of natural [51] or cultural heritage [52,53]. Moreover, numerical models of temperature distribution are useful for detecting the existence of thermally driven deformation comprising both quasi-cyclical (reversible) and irreversible (plastic) strains [54].

**Figure 1:**
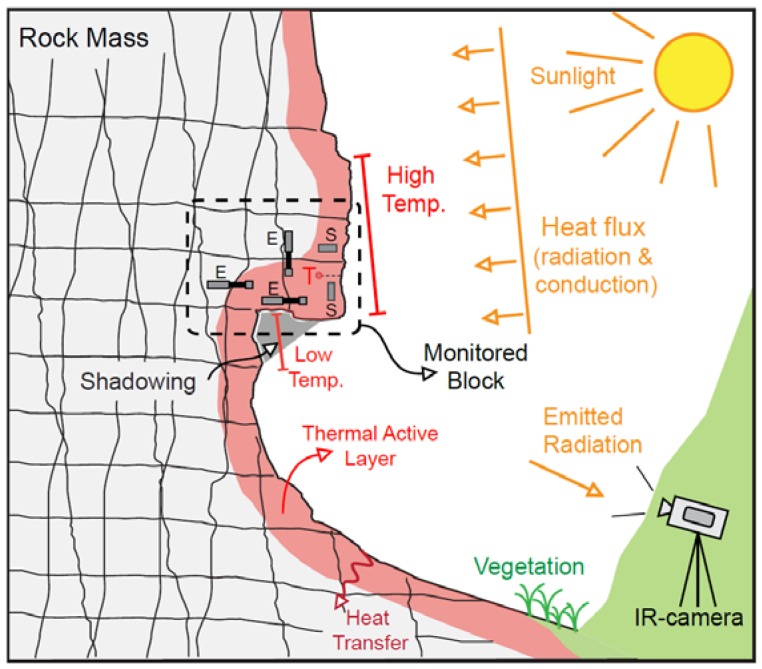
Sketch of the solar radiation effect in the instrumented rock-block at selected test-site. Heat flow propagates as cyclical input within the thermal active layer, influenced by irregularities of the surface, exposure, shadowing effects and presence of moistened or vegetated zones (modified after Wu et al. [29]).

In order to quantify the effect of intense solar radiation on discontinuous rock mass and evaluate the predisposition of the latter to thermally-induced strains, a two-year thermal monitoring was monthly carried out on a selected test-site where an intensely jointed rock-block is exposed to direct solar radiation (Figure 1), through integrated direct and indirect (i.e., InfraRed Thermography—IRT) sensing techniques, by the combined use of a rock thermometer and a thermal camera.

For the considered case studies, the control of rock face orientation (aspect) and the role of joints in heat propagation within the rock mass have been studied by analysing daily and seasonal temperatures variations in the rock mass on both its surface and in depth, adopting 1D and 2D analyses. A conceptual model for the development and progression of thermally induced strains has been also proposed. Basing on this model, an experimental approach for estimating the predisposition of a rock-wall in a quarry area to thermo-mechanically driven rock-blocks instability have been tested.

## 2. The Acuto Quarry Test-Site

**Figure 2:**
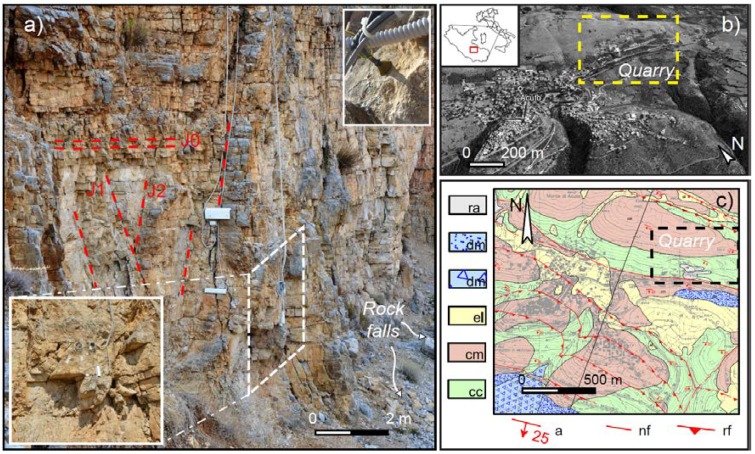
(**a**) View of monitored rock-block with respect of the stable rock wall behind. The rock mass is pervasively jointed by primary and secondary discontinuities. Details of the deformative monitoring system are also shown in the inset picture on bottom-left. The frontal face of the block is also instrumented with a rock thermometer; (**b**) satellite view of the Acuto quarry; (**c**) geological map of Acuto area (credits P. Sarandrea, 2008): made ground (ra); slope debris (dm—Holocene); eluvial-colluvial deposits (el—Holocene); Cenozoic limestone (cm—Serravallian—Langhian); Mesozoic limestone (cc—Campanian—Coniacian); attitude of beds (a); normal fault (nf); inverse fault (rf).

The Acuto test-site is located NE of Acuto municipality on Mt. Ernici ridge (central Italy), where Mesozoic limestones (mudstone or floatstone with rudists) widely outcrops (Figure 2). An abandoned quarry was selected to host a monitoring system devoted to understanding the thermal response of rock mass at rock-block scale. For a climatic classification of the study area, temperature and rainfall data acquired over the past 15 years from the closest weather station of the Piglio municipality (located 5 km away from test-site) were considered. The average monthly temperature trend presents a maximum net for the months of July (32.8 °C) and August (32.6 °C), while the coldest months are January and February with average minimum air temperature of 1.5 °C, characteristics of a temperate climatic region. The average monthly rainfall presents a maximum of 120–130 mm at the Autumn-Winter transition while presenting a minimum of 38 mm in the period ranging between July and August, concurrently with the maximum air temperature.

The N20E trending quarry wall is exposed to intense solar radiation and predisposed to evolve through gravitational instabilities by rock fall, sliding or toppling mechanisms. Moreover, the quarry rock wall is characterised by heights ranging from few meters up to 50 m and a length of hundreds of meters. The rock mass is also intensely jointed: geomechanical scanlines and remote surveys using total station have been performed on the quarry wall and five joint sets were distinguished [55], a S0 (93/4; dip direction, dip) system, corresponding to the limestone strata, S1 (131/82), S2 (91/64), S3 (4/80) and S4 (198/86).

Rock samples were also collected and tested in the laboratory to derive physical thermal properties of the rock matrix. In order to constrain the heat propagation in the rock medium, thermal conductivity tests were thus performed, according to ASTM E1530-11 standard by guarded hot plate method. The obtained results deriving by test on two prismatic samples (150 × 150 × 70 mm) showed a thermal conductivity value of 2.09 ± 0.01 W/mK.

A predisposed jointed rock-block 20 m^3^ sized was selected as main target for the installation of a monitoring system in order to understand its thermal response under direct solar radiation and the heat propagation in 2D conditions (Figure 2a) [56]. The block is isolated from the rock wall behind and protruded with respect to the quarry face by opened joints and exposed to sun radiation along two faces: a front face exposed to the East and a back face exposed to the South.

The multi-sensor thermal monitoring system consists of a rock thermometer (Type-K Thermocouple), installed at the centre of the monitored rock-block, in the front face at a depth of 8 cm from the surface, to measure the inner rock mass temperature and of a weather station, installed at the top of the slope, equipped with air-thermometer, hygrometer, rain-gage and anemometer for wind speed and wind direction. All sensors are set to acquire data every minute with storage in a local data-logger CR1000 Campbell Scientific, equipped with 24 acquisition channels. The data are transmitted to a local server, every 4 h, by a GPRS wireless connection system that enables remote control of datasets.

## 3. IRT-Survey: Principles and Methods

The multi-parametric monitoring system has been implemented with seasonal remote surveying via InfraRed Thermography (IRT) in order to evaluate the role of both solar radiation and rock-block exposure on surficial rock temperature. IR-imaging represents a reliable, rapid and non-destructive technique that allows to derive temperature distribution at the rock-air interface, quantifying the effect of solar radiation on heat propagation over a discontinuous medium [57,58].

**Figure 3:**
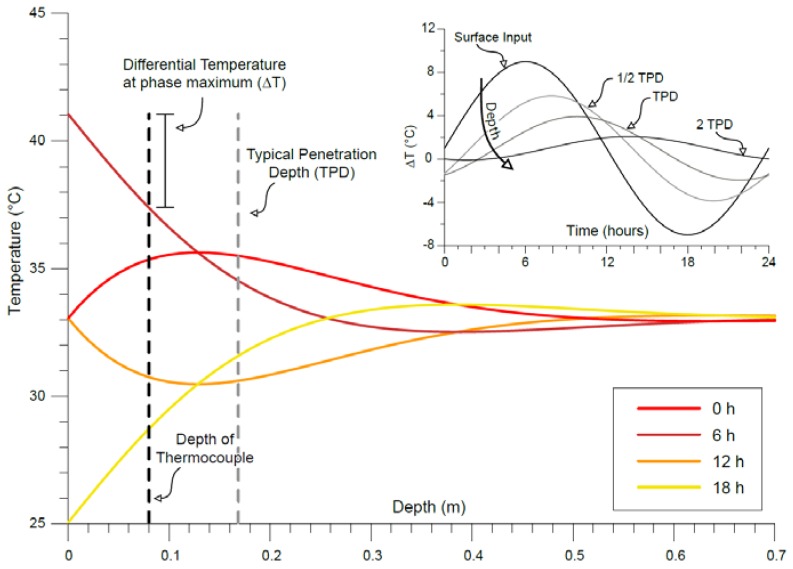
Theoretical periodic heat propagation in depth at different phases (in hours) obtained following Carslaw and Jaeger [59]. The typical penetration depth (TPD) in the Acuto test site corresponds approximately to 17–18 cm. The inset graph shows the different amplitude of thermal perturbations for different multiples or fractions of TPD.

Rock masses are exposed to daily and seasonal temperature variations at its surface, which propagate in depth according to the Carslaw and Jaeger [59] model, which describe in a periodic state the temperature (*T*) profile in depth (*x*) for a semi-infinite solid:

(1) $$T\left(x,t\right)=\;T_{0}+Ae^{-x/\sqrt{2a/\omega }}\cdot sen\left(\omega t-x/\sqrt{2a/\omega }\right)\;$$

where the term $\sqrt{2a/\omega }$ represents the decay factor describing the exponential decrease of the thermal wave amplitude with depth (x, Figure 3). This factor is controlled by rock thermal diffusivity (a), which is a function of rock density, thermal conductivity and heat capacity of the rock medium. This term corresponds to a typical depth of penetration (namely TPD, Figure 3) [12] of the periodic thermal front within the solid, that is, a thermally active layer (approximately 20–30 cm deep) where the surficial perturbation propagates.

In order to understand the amplitude of thermal perturbation and assess the role of cyclical forcing in the stability of rock masses, a combined use of direct and indirect sensing techniques has been adopted availing of the type-K thermocouple (hereafter named rock thermometer) and a TESTO^®^ 885-1 thermal camera (measuring range between −30 and 100 °C; accuracy of measurements ±2%; FOV 30° × 24°; image resolution 320 × 240 pixel; maximum geometric resolution of 1.7 mrad). With the aim to validate the IRT technique and verify the accuracy of the obtained results, a comparison between rock temperature in depth (derived by rock thermometer) and on its surface (derived by IRT) was performed.

Thermal monitoring was carried out with a seasonal cadence over two years, trying to constrain the impact of sunlight radiation and the impact of opened joints on heat propagation across a jointed heterogeneous medium. The thermal monitoring was achieved selecting days presenting the same weather conditions and choosing preferably sunny days without cloud cover, to minimise the daily variability and highlight the seasonal signals. It worth note that all the timing hereafter discussed are referred to solar times.

**Figure 4:**
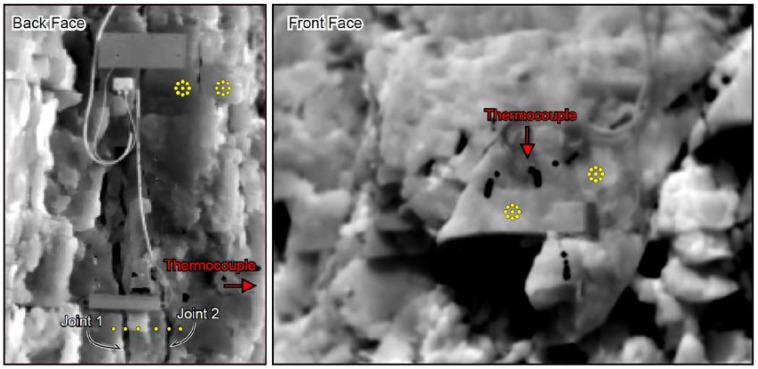
Location of temperature control points on the back (**left**) and front (**right**) face and across the main opened joint sets (**left**). The locations of the rock thermometer are also shown by red arrows for both views. The reader is referred to the squared 30 cm length carter for the scale.

The instrumented rock-block, protruded with respect to the rock wall behind, was selected as the target of the IRT analyses (Figure 1 and Figure 2), which were carried out from two different points of view, framing frontal and back faces of the rock-block (Figure 4), characterised by a different solar exposure.

The monitoring was performed by means of standard acquisition of thermal images over the diurnal hours (from 8:00 to 17:00) with a regular time-interval of 30 min, framing the block from a distance of about 5 m apart from both faces of the monitored rock-block. Such a distance is small enough to guarantee a spatial resolution of about 8.5 mm and to separately detect the largest set of joints. The total acquisition was designed to avoid perturbative effects related to distance and angularity of the target from the point of view [60,61] as well as parasite radiations.

Emissivity values of rock mass was evaluated a priori adopting a standard reference method with the use of reference material with a known emissivity (ISO 18434-1). Field measurements of reflected apparent temperature were carried out using a reflector method, according to the ISO standard, availing of aluminium foil and setting an emissivity equals to one and a distance between the camera and the monitored object to zero.

For each thermal image, environmental controlling factor such as air temperature and humidity [62] were also settled in the thermal camera proprietary open-source software TESTO-IRSoft^®^, which was derived for each IR-measure by weather station acquisition.

A quantitative analysis of thermal images was carried out through the extraction of thermal time-series from some rock mass zones selected on both rock-block faces. To this aim, thermal images were converted to thermal matrixes from .csv to .ASCII file format by the proprietary software and a Phyton compiled script. Thermal time-series were extracted by selected control points on the georeferenced images for different zones of the rock mass. To guarantee a statistical significance of the recorded thermal values and filter local temperature difference resulting by surface irregularities and asperities, the derived temperatures were averaged by measuring on the nine neighbour control points in the two rosettes (yellow dots on Figure 4). Differential temperature obtained between the two rosettes of control points, under homogeneous solar radiation, remain confined within the standard deviation, revealing limited influence of surface asperities on the monitored faces.

In order to evaluate the feasibility and representativeness of the passive IR monitoring techniques the surficial temperature distribution derived by thermal camera was compared with temperatures directly derived by the rock thermometer installed in the rock matrix and deconvolved to the surface according to the semi-infinite heat propagation law described by Equation (1). The IRT approach was also tested for the monitoring of temperature distribution across main open joints located on the back face of the target rock-block (Joint 1 and Joint 2 in Figure 4), which isolates the rock-block from the quarry wall behind, in order to investigate the role of rock jointing conditions in the heat propagation and evaluate its influence in the temperature distribution within the jointed medium.

This sensibility test was performed to understand the role of joints in a 2D heat propagation model affecting a rock mass. Two main joints, with an average opening of about 5 cm were selected on back face of the rock-block at 50 cm and at 70 cm far from to the front face of the rock-block (Figure 2a and Figure 4). In correspondence to these two discontinuities, cross sections of temperature values astride the main joints were output over the whole monitoring period, by sampling temperature values from IR-images inside and outside the trace of the joint (Figure 4).

**Figure 5:**
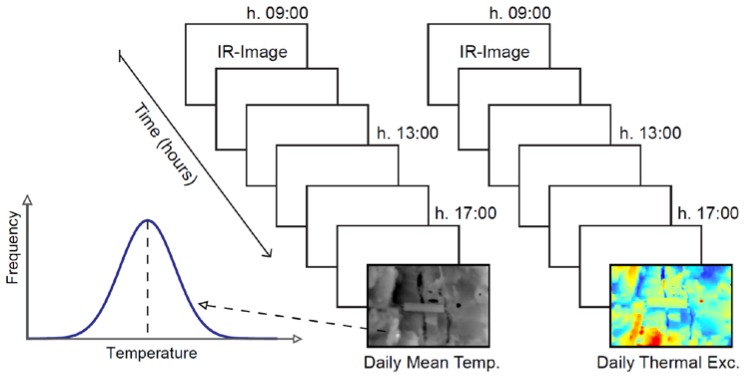
Method of Image processing adopted for the 2D-analysis. Daily Mean Temperature with relative daily variability (expressed by St. Dev.) pixel-by-pixel were calculated. With the same approach, the spatial variability of Daily Thermal Excursion was defined.

Thermal images collected throughout the 2-years were also used for testing 2D thermal analysis at the rock-air interface. This analysis is devoted to the comprehension of spatial temperature distribution on the quarry face across a discontinuous or inhomogeneous medium. Daily mean temperature and daily thermal excursion were calculated for every cell of the raster images, combining pixel-by-pixel the standardised thermal images acquired each 30 min by the IR-camera, by simple “Mean” and “Range” image processing functions (Figure 5). These analyses were achieved only for the back face of the monitored rock-block. The adopted 1D and 2D approaches can respectively represent a standard practice either for the monitoring of single rock-block or for the monitoring of wider sector of rock masses.

## 4. Results

### 4.1. Daily Thermal Behaviour of Rock-Block by 1D Analysis

**Figure 6:**
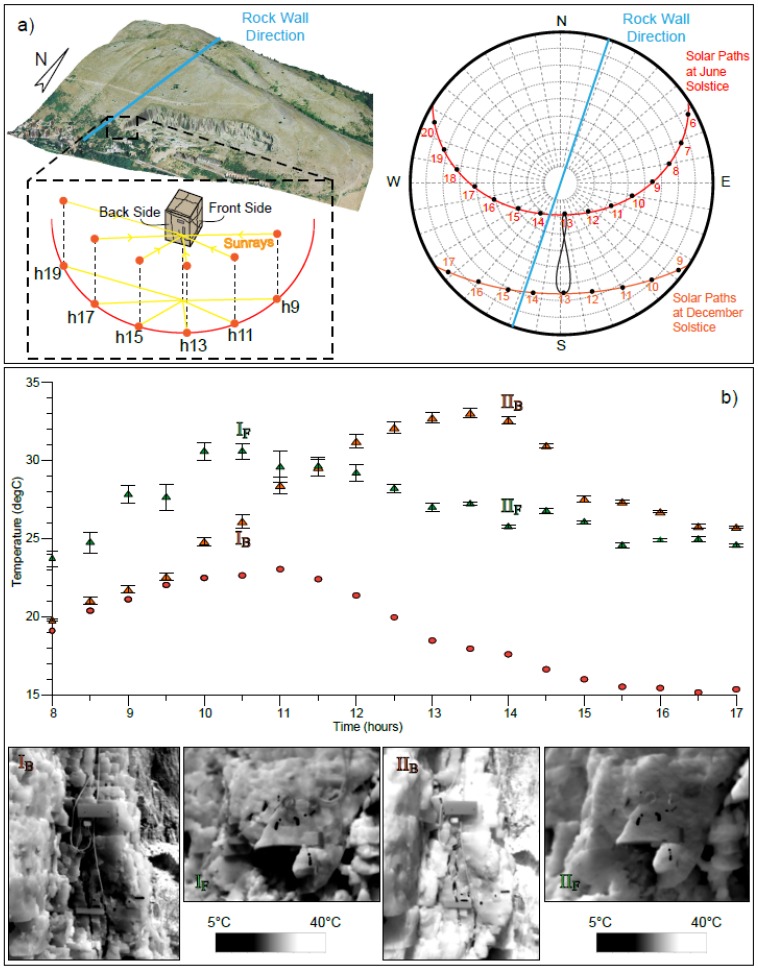
(**a**) Position and exposure of the rock-block respect the N-S trending quarry face and to the incident sunrays. A daily and seasonal variability of solar paths is reported by local solar chart. The different exposure reflects in an out-of-phase peaks between the two faces of the rock-block (**b**), which, because of its orientation, appear alternatively sunny or shadowed. A comparison of surficial temperatures with the rock thermometer (red dots) and example of related IRT images are also shown.

The use of IRT techniques allowed to derive surficial temperatures at the rock-air interface of the target rock-block describing its heating and cooling phases, from 8:00 to 17:00 for each monitored. The monitoring allowed to recognise the surficial rock temperature distribution inferring the diurnal part of the thermal cycle. In each daily thermal cycle, a first heating phase is followed by a slower cooling phase (Figure 6b), controlled by heat transfer processes including both thermal conduction and convection of the air masses, as well as direct solar radiation. The first phase starts at the sunrise and ends at midday, as a result of direct solar radiation. The second phase occurs during the progressive shadowing of the quarry wall in the afternoon and night (stage II_F_ on Figure 6). More in particular, the cooling phase is divided in two parts: (i) a first one immediately after the rock shadowing, characterised by a fast cooling resulting from the removal of the thermal perturbation (i.e., before the sunset); (ii) a second one, occurring during the night time, characterised by a slower heat loss (well described by the rock thermometer series—Figure 6b), towards isothermal condition with the surrounding environment. This second stage is driven by heat exchange between the rock mass and the external air temperature, according to a cooling rate function of rock thermal heat capacity (Figure 6b). The inflection point of the cooling phase occurs approximately at 13:00 for the front-face and at 15:00 for the back-face.

**Figure 7:**
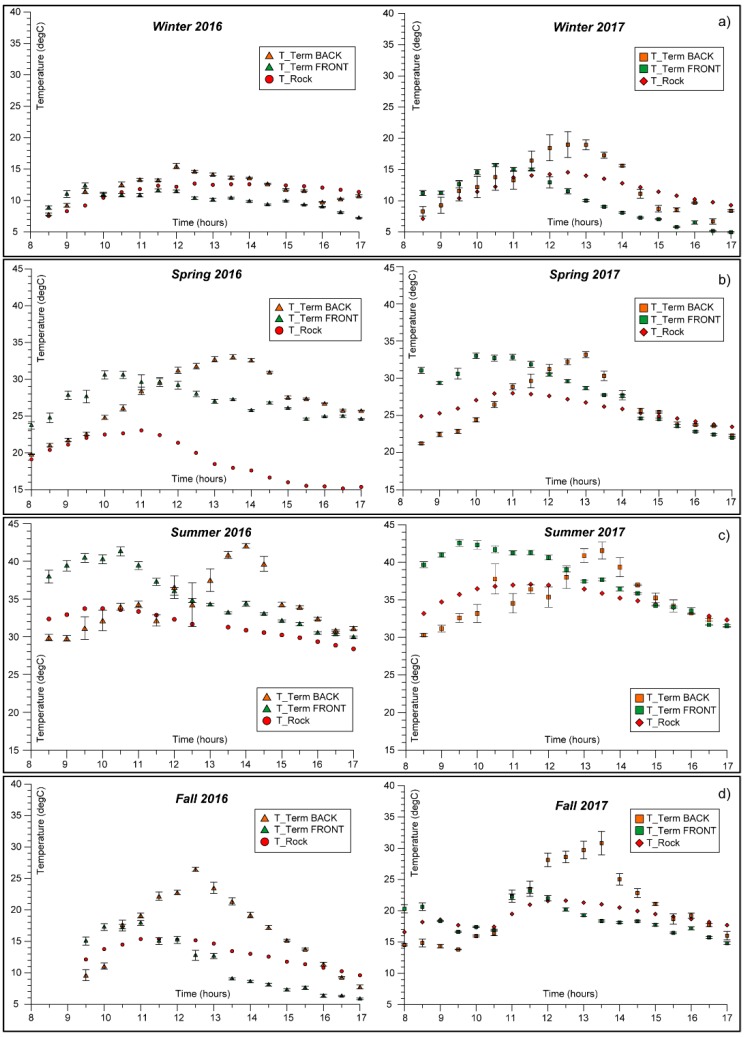
Daily and seasonal variability of surficial (T_Therm) and inner (T_Rock) rock temperatures resulting by IRT and direct sensing techniques respectively, derived along frontal (green points) and back (orange points) faces of the rock-block. The rock thermometer temperature values are reported by red markers.

The analysis of temperature time-series highlights how heating phases are always shorter than the related cooling phases due to effect of short, direct and intense solar radiation until noon. In fact, the rock heats up only during the hours of direct solar radiation, resulting in a fast rise of surficial temperature which affect the lone diurnal stage of the day. The resulting temperature time-series (Figure 6 and Figure 7) also evidenced the role of solar radiation in the heating of the rock mass, showing a clear dependency of thermal input on both sun paths and rock-block exposure. The two faces (front face and back face) of the rock-block, in fact, have an orthogonal exposure thus reaching maximum values of the temperature at deferred time, showing out of phase peaks.

The Eastward front face of the rock-block is irradiated by solar light since dawn to midday, while the back face becomes lighted up since the first hours of the afternoon until sunset because of its Southern exposure. It follows that the rock-block experience a continuous heating across its faces, showing a front face and back face reaching their maximum temperatures at 10:30 and 14:00, respectively (Figure 6a,b).

Thermal images acquired using IRT technique during the beginning of the heating phase show a higher temperature variability at the surface of the rock mass if compared to the intermediate and final phases of the same heating process. In the thermal images acquired in the early hours of the day (about at 8:30–9:00), several isothermal bands are identified describing an inhomogeneous distribution of rock temperature on both faces. The presence of these isothermal bands is attributable to differential heating affecting the rock-block because of an irregular geometric shape of both rock faces and angle of incidence of the sunrays, which changes according to different positions of sun during its daily solar path (Figure 6a). When the incidence of sunrays is perpendicular to both the block faces (at 10:30 for front face and at 14:00 for the back face—points I_F_ and II_B_ on Figure 6) the perturbation caused by the geometric irregularities of the rock surfaces decreases and the surficial rock temperature tends to become uniform reaching a maximum peak value (Figure 6b).

The timing of the temperature maximum appears to be shifted in the daytime with respect to seasons, evidencing anticipated timing of the temperature peak on front face and a delayed thermal maximum on the back face, moving from Winter to Summer (solstices on Figure 6). This effect can be regarded as a consequence of solar path variation and sun elevation over the year. (Figure 6a). The seasonal variability of thermal input will be detailed in the following sections.

Furthermore, the IRT monitoring data of the front face can be directly compared with the rock thermometer records to evaluate the effect of direct solar radiation, describing the heat propagation in depth and evaluating the temperature decay with depth (evidenced by temperature difference between the surface—green triangles relative to front face—and the inner rock temperature, marked by red circles—on Figure 6b).

### 4.2. Seasonal Thermal Behaviour of Rock-Block by 1D Analysis

Yearly thermal variations were also recorded over the monitoring period, due to seasonal temperature changes related to local climatic conditions. The comparison between daily temperature series over all the considered period highlighted magnitude, amplitude and the seasonality of the thermal input influencing the rock mass (Figure 7).

To analyse the thermal behaviour of the rock-block exposed to the sunlight, a description of the half-day thermal cycle of surficial rock temperature (on both front and back faces) and inner rock temperature at a depth of 8 cm was achieved for every monitored season during the period 2016–2018.

As shown in Figure 7, an analogous behaviour over seasons, characterised by similar absolute temperatures values and timing of thermal maxima. The minimal differences founded among months belonging to the same season are attributable to episodic variations in local meteorological conditions and not to different climatic factors (e.g., initial flat portion in Autumn 2017 of Figure 7d due to a short persistence of cloudy cover).

The IRT monitoring campaigns during the local temperate Winter seasons (Figure 7a), when the direct solar radiation is lower, allow to derive that the rock-block in its front face experienced a maximum temperature up to 15 ± 0.47 °C, reached between 10:00 and 10:30. Starting from 11:30, the surficial rock temperature of the front face decreases and remains below the rock temperature detected by rock thermometer for all cooling phase, testifying the immediate and rapid cooling at rock surface since the insolation halts and the shadow involved the whole block (II_F_ on Figure 6d). On the back face, a maximum surficial temperature value of 19 ± 0.42 °C is reached around 12:30. The maximum temperatures value recorded by IR-camera on the front face is about 4 °C higher than the ones detected by the rock thermometer at depth of 8 cm. After 14:30, because of the exposure and the height of the rock wall, the whole block overshadows and the curves reverse, causing a heat release from the centre of the block towards the outside.

During the Summer (Figure 7c), when direct solar radiation is more intense, the rock-block in its front face experienced a maximum temperature up to 43 ± 0.45 °C. As described above, this peak is reached around 09:30 (Figure 7c) because of the higher elevation of the sun paths. The maximum temperature values recorded by IR-camera on the front face at its peak is about 8 °C higher than the ones detected within the rock by thermometer. On the back face, a maximum value of 42 ± 0.32 °C is reached around 13:30–14:00 for both the monitored years, reaching more than 10 °C of difference respect to the rock thermometer, which, because of its position within the front face, is already undergoing a cooling phase (Figure 3).

A comparison between temperatures obtained on surface and in depth by the two techniques (IRT and Thermometry) can be performed only for the frontal face. In Summer, the intense radiation warms the block for all the afternoon, delaying the thermal inversion to the night hours, as evidenced by the position of the IRT temperature curves (green and orange points in Figure 7) located above the rock temperature curves (red points in Figure 7) until the 17:00.

Spring and Autumn (Figure 7b,d) reveal more variable trends of temperature. The IRT monitoring performed during the Spring shows that the front face of the rock-block experienced maximum temperature values ranging from 30 up to 33 ± 0.43 °C; similar maximum values (i.e., up to 34 ± 0.35 °C) were reached on the back face.

During the Autumn, the front face of the rock-block experienced a maximum value of 23 ± 0.6 °C in sunny days, which appear considerably lower than the maximum temperature experienced on the back face at its peak, when about 31 ± 1.87 °C was reached. This marked temperature difference is ascribable to the more pronounced effect of the solar radiation during Autumn, which affects a rock mass initially colder than what observed in warmer seasons.

The maximum difference between the surficial temperature on front and back face and the internal rock temperature is reached in Spring and Summer when the solar radiation is highest.

To assess the validity of the IRT technique for the evaluation of temperature distribution along a wide rock face, a comparison of the surficial temperature values derived by IRT with respect to the theoretic values derived by rock thermometer and deconvolved to the surface was performed, performing an analytical computation for a semi-infinite solid domain [59].

**Figure 8:**
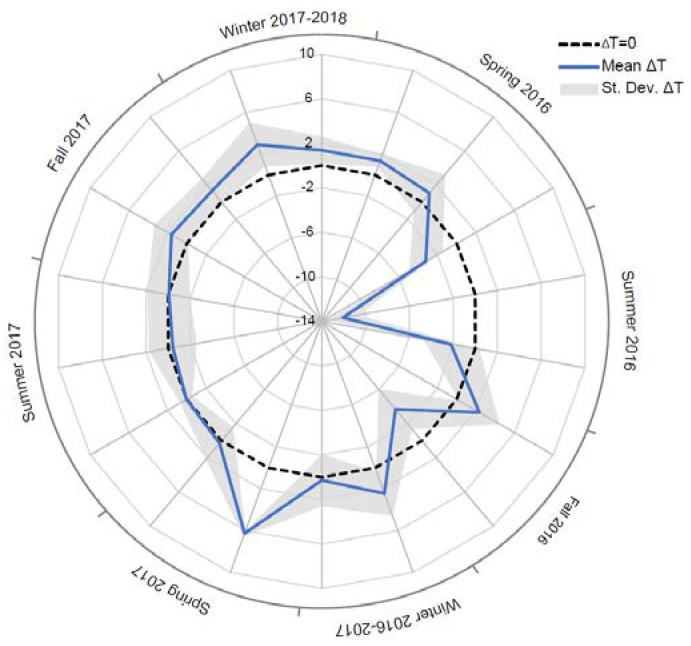
Radar-plot of temperature difference (ΔT) between the surficial rock temperature (remotely derived by IRT) and the deconvolved thermal signal directly measured by rock thermometer and reported to the surface in the overall monitoring period. The dashed black line indicates the perfect agreement between the two techniques and the optimal thermal resolution of the IR-sensing. The maximum error bar introduced by the IR-technique is shown by the grey crosshatch.

The comparison between the two temperature datasets derived by direct and indirect sensing highlights a good agreement between the two different techniques, showing a temperature difference generally enclosed within a maximum ±2 °C, represented in the radar plot by the difference between the blue line and the black dashed line (Figure 8). These gaps are locally out of the range of sensibility of the IR-camera (±2%), which could be also include systematic and random errors and surface irregularities effects. Episodic large discrepancies, reported in Figure 8, were attributed to variability of the weather (and temperature) conditions during the days before the execution of the thermographic survey.

Based on surveyed surficial rock temperature, seasonal heating and cooling rates of the interface have been calculated for the back face of the rock-block, in which, because of the time at which the peak is reached, the heating and cooling ramp have the same duration. The surficial rock thermal rate, derived by the temperatures extracted under 1D conditions by the rosettes of points in Figure 4, is described as follows:

(2) $$TR_{s}=\;\frac{∆T_{s}}{∆t}$$

where $∆T_{s}$ is the variation of surficial temperature values between 09:00 and 12:00 in case of the heating ramp (HTRs) and between 14:00 and 17:00 in case of cooling ramp (CTRs), while ∆t is the considered time interval. In order to study the cooling behaviour of the rock mass the complete shadowing of the rock faces is required. The rates were calculated monthly and then averaged to return seasonal values.

The analysis of heating and cooling rates allows to derive peculiar features in the analysis of rock thermal behaviour: in the Summer season, the rates showed the same value for both heating and cooling phases, describing a slow thermal variation of about 1.90 °C/h (Table 1). In fact, the thermal excursion experienced in the three hours considered for the calculation of TRs (both HTRs and CTRs), has lower values than those of the other seasons because of the greater uniformity of the solar radiation and to the higher initial level of air temperature. The season with the highest thermal rate is the Autumn, which, due to the greater thermal excursion able to reach 8 °C in the cooling ramp and 9.3 °C in the heating ramp, entail a cooling rate of 2.88 °C/h and a heating rate of 3.59 °C/h (Table 1). In fact, in the Autumn season, the direct solar radiation is high and cause a rapid heating of the rock during the considered hours, which affects a rock interface cooled by air with a lower temperature.

The thermal excursion recorded in Winter and Spring, presents value with higher variability ranging from 5.4 °C to 7.5 °C that entails an average heating and cooling rate of 2.5 °C/h (Table 1). Despite the high radiation of the sun in Spring season, this perturbation involves a rock mass which already starts from higher temperature, derived by an initial equilibrium with warmer air masses than the ones in the Fall season. The analysis of the CTRs, instead, reveals a more homogeneous behaviour (Table 1), which, because of the lack of thermal perturbations (i.e., after shadowing or sunset), appears mostly controlled by the stored heat by the rock mass and by its thermal capacity.

The seasonal values of surficial rock temperature rate and the thermal excursion measured by IRT at the back face of the rock-block are summarised in Table 1.

Moreover, for the front face, only a seasonal cooling rate was calculated because of the narrow time-lapse occurring between the first IRT daily acquisition and the temperature peak (reached around 11:00). After this peak, in fact, the rock-block is shadowed and the surficial temperature gradually decreases.

The thermal excursion in cooling derived for the front face confirms the higher values in the half seasons, which reached values of 3.75 °C in the Spring and of 5.26 °C in the Autumn (Table 2).

Thermal excursion over Summer season is just 1.95 °C, while in Winter season it assumes maximum values of 1.60 °C. Also at the front face, the cooling rate of the surficial rock surface was calculated monthly and then averaged to return a seasonal value, taking into account a different time interval between 12:00 and 15:00 (i.e., three hours after the respective temperature peak), using the same equation presented before.

The cooling rates for the mid-seasons are higher than ones derived in Summer and Winter (0.65 °C/h and of 0.54 °C/h respectively), as previously observed for the back face. Nevertheless, their absolute values are sensibly lower than ones obtained for the back face, due to the different timing during the cooling rate has been calculated. Because of the occurrence of the cooling of the front face in a time-lapse between 12:00–15:00, when a contemporary increase of air temperature takes place, a direct comparison between the rates derived by the two faces cannot be carried out.

Specific seasonal values of surficial rock cooling rates and thermal excursions in the cooling ramp for the front face of the rock-block are summarised in Table 2.

The IRT monitoring is also valuable to derive the annual thermal behaviour over the four seasons, highlighting the difference between warm and cold periods and inferring long-term thermal cycles.

**Figure 9:**
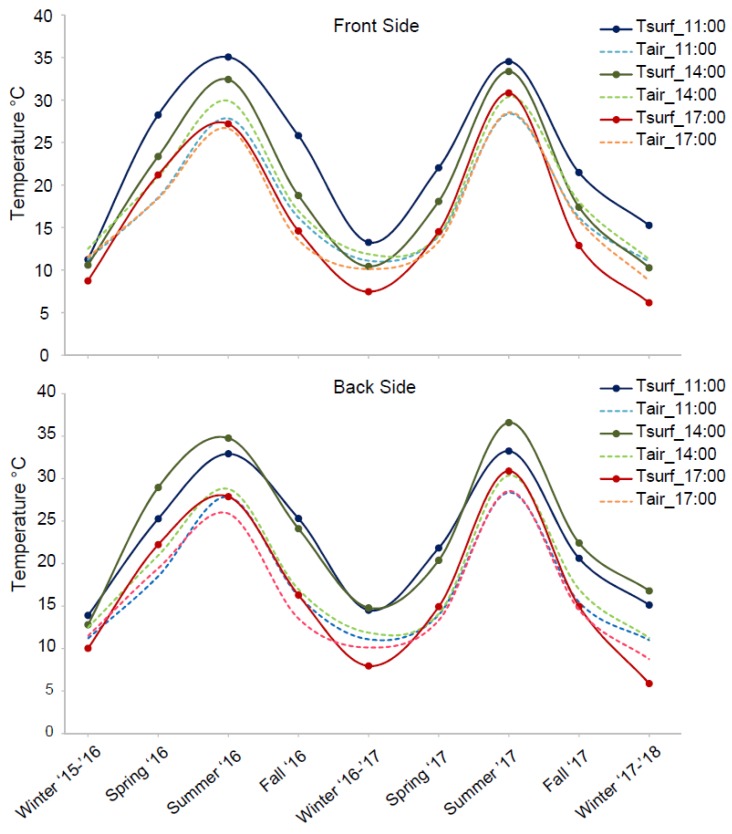
Annual temperature trend for the front- (**top**) and back face (**bottom**) of the rock-block at different time intervals.

At this aim, the temperatures at rock surface over the two years were evaluated for the front and the back faces of the rock-block, taking into account three different hours (Figure 9): (i) 11:00 when the temperature peak was reached on the front face, (ii) at 14:00 when the temperature peak pertains the back face and (iii) at 17:00 when the shadow involves entirely the rock-block. For all seasons, the value of the surficial rock temperature is calculated by averaging the values monitored over the individual months belonging to each season, with the purpose to recognise the long period thermal wave filtering the single-day variability.

The annual temperature distribution for each hour (Figure 9), allows to qualitatively derive the annual temperature trend, which involves the rock mass and to detail the warming and the cooling ramps typical of Spring and Autumn, as well as the annual mean values of maximum and minimum temperatures. More in particular, the thermal excursion in the seasonal cooling phase between Summer ’16 and Winter ’16–’17 is about 22 °C while between Summer ’17 and Winter ’17–’18 is about 20 °C. The thermal excursion in the seasonal heating phase between Winter ’15–’16 and Summer ’16 is about 24 °C, while between Winter ’16–’17 and Summer ’17 is about 22 °C. These values of seasonal thermal excursion suggest the semi-stationarity of the thermal input over the two monitored years, allowing to derive the amplitude of the annual thermal perturbation that affected the rock surface.

The comparison with the annual air temperature revealed how maximum differences between the surficial rock temperature and the air temperature are reached on both faces, at time intervals in which the incident solar radiation is higher (i.e., at 11:00 for the front face—blue line on Figure 9 and at 14:00 on the back face—green line on Figure 9). These differences tend to decrease when both faces become involved by the shadow (from 14:00 and 17:00 for front face and back face, respectively), as a direct effect of the solar radiation. The role of this agent is confirmed by the comparative analysis of these differences over the all seasons, which demonstrate a greater amplitude in Summer period with respect to the other season for each of the time-intervals considered. Because of this lower solar radiation, the inversion of the rock temperature with respect to the one of the air, occurs only in Winter season at time intervals function of the exposure.

The IRT survey was also applied to the back face of the rock-block, which, when not exposed to direct radiation, can be considered as a natural cross section perpendicular to the quarry face (i.e., front face). On this side, the temperature distribution across two main opened joints was evaluated (Figure 4), testing its thermal response to solar input and the temperature decay in depth with respect to the front face. It is worth note how these joints are positioned at depth greater than the TPD respect the front face (Figure 4).

**Figure 10:**
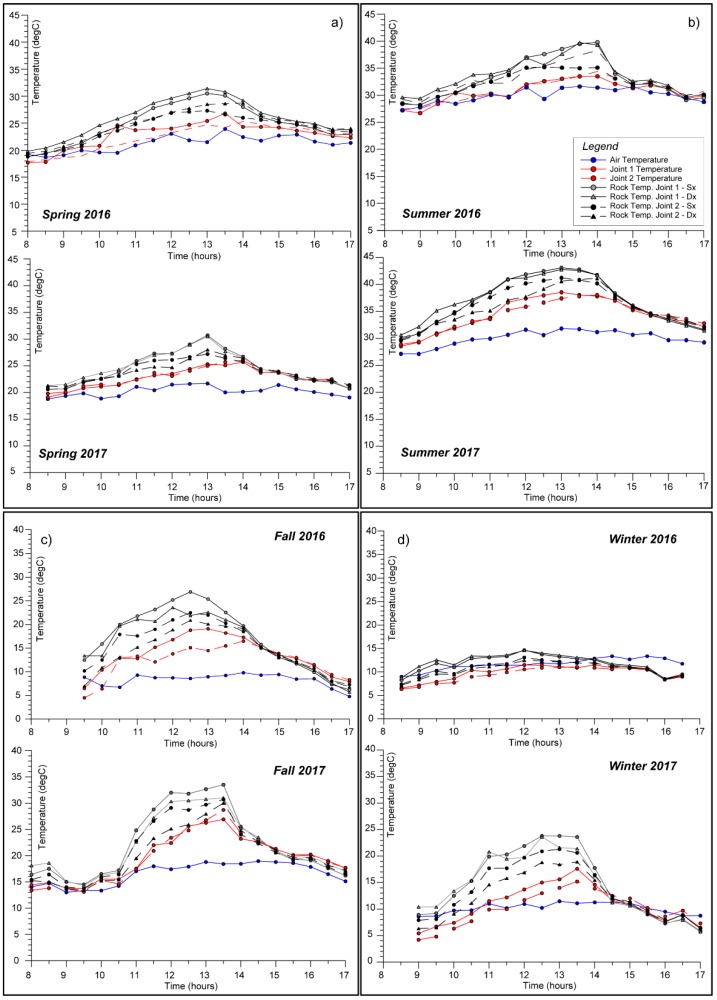
Daily and seasonal variability of surficial rock temperature across joints (dark lines), air temperature filling the joints (red lines) resulting by IRT sensing techniques. The environmental air temperature series derived by weather station are reported by solid blue line.

During the heating phase of Spring and Summer, the air temperature within the joint is almost 5–6 °C lower than the surrounding rock temperature, because of the difference in thermal conductivities and specific heat capacities between the two media. This temperature difference can reach 8–10 °C in Autumn and Winter seasons, due to the lower temperature of the air filling the joint. During the cooling phase of all the four seasons, the air temperature within the joints and the rock surficial temperatures gradually decreases towards the same value, reaching isothermal conditions. The air temperature is consistently lower than the one founded within the opened joints, so revealing an influence of the warmer rock on the air temperature within the joints (Figure 10). More in particular, during the cooling phase the rock mass around the joints releases heat tending to warm up the temperature of the air by a radiative process.

No significant temperature decay was observed across the two joint-walls of the opened joints (Sx and Dx of Joint 1 and Joint 2 in Figure 10) during the heating phase, because of their large distance from the heated surface (i.e., front face) and their position outside the related TPD (Figure 3) where slight temperature changes occurred. In addition, given their exposure to the sunlight in the rest of the day (until 14:00 during the heating of the back face—Figure 6), no detectable contrasts were observed in the two joint-walls.

### 4.3. Thermal Behaviour of Rock-Block by 2D Spatial Analysis

The results of the 2D analysis conducted on the overall thermal images derived by daily IRT surveys, evidenced a Gauss-normal spatial distribution of temperatures in the framed rock face. The modal values of mean temperatures of the four seasons showing a minimum in Winter and a maximum in Summer with a more pronounced spatial homogeneity in the first case, as evidenced by the lowest standard deviation. The result (Table 3) of the spatial distribution of daily thermal excursion highlights the minimum absolute value and the minimum dispersion in the Winter season, where 6.00 ± 1.13 °C were derived. On the contrary, in Autumn the 2D analysis supports the conclusions of the 1D analysis performed on the selected rosettes of points (Figure 4), in which the maximum thermal excursions in the monitored half-cycles were observed and where, for some portion of the rock-block, more than 16 °C of temperature variations were experienced. This value is consistently higher than the ones returned from 1D analysis (Table 1 and Table 2), where the effects of the exposure have been not perceived.

**Figure 11:**
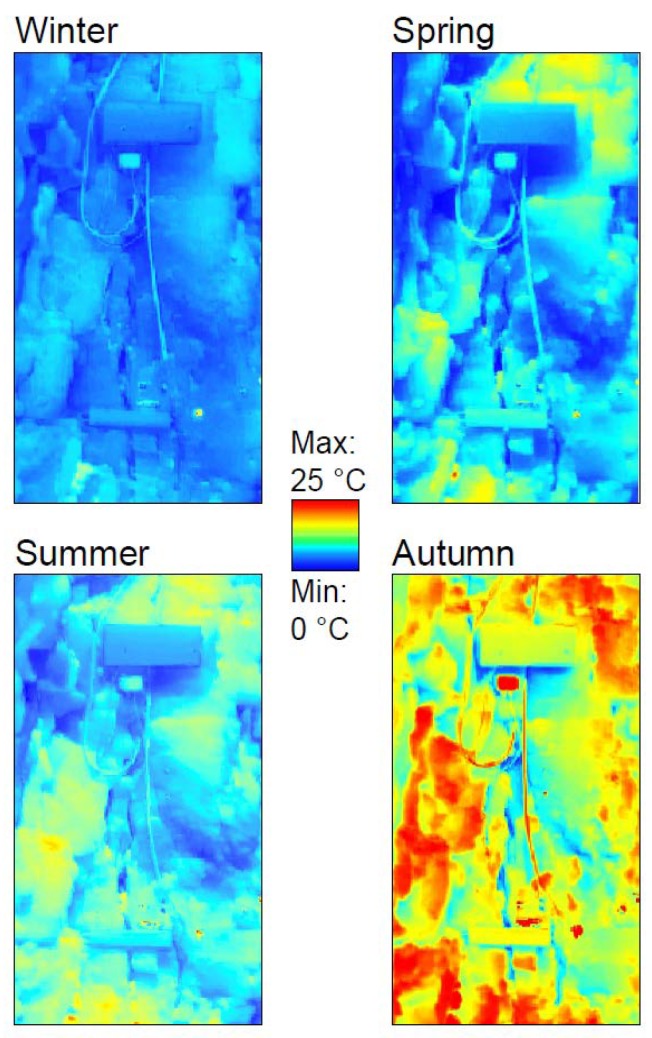
Spatial distribution of Daily Thermal excursion acquired in the four monitored seasons. False colour images were obtained applying the multi-temporal 2D analysis carried out on the georeferenced IR images.

**Figure 12:**
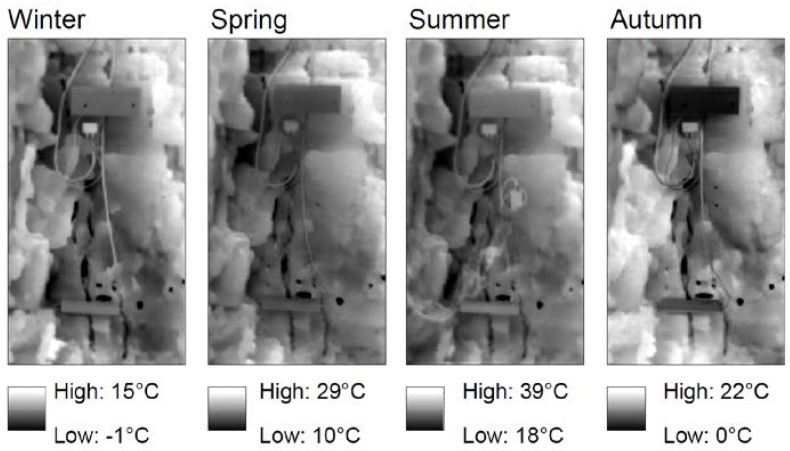
Daily Mean Temperature distribution derived by multi-temporal 2D analysis in the four monitored seasons.

The 2D multi-temporal analysis carried out on all daily IR images acquired in the four seasons allow to derive pixel-by-pixel data on spatial distribution of Daily Thermal Excursion (Figure 11) and Daily Mean Temperature (Figure 12) suffered by the whole back face. As regarding the daily thermal excursion (Figure 11), the Winter season present low and homogeneous values on the considered surface. This evidence is ascribable to the low intensity of solar radiation and to the low air temperature values that characterise Winter season. Even in the Summer season low values of the daily thermal excursion were suffered by the rock surface, due to the very high solar radiation and the high value of air temperature, which do not allow the occurrence of large temperature excursions over the monitoring time. Opposite results were obtained in Spring and in the Autumn season, when very high daily thermal excursion occurred in many portions of the back face which experienced values up to 25 °C. These higher values are due to important thermal difference between day and night and to very intense solar radiation.

With the same approach, the Daily Mean Temperature suffered pixel-by-pixel by the back face was obtained (Figure 12). In this case, the spatial variability of the result is very high, presenting maximum and minimum values directly correlated with the seasonality of the radiative input, with highest values in Summer and minimum temperature, locally below 0 °C, in the Winter season.

**Figure 13:**
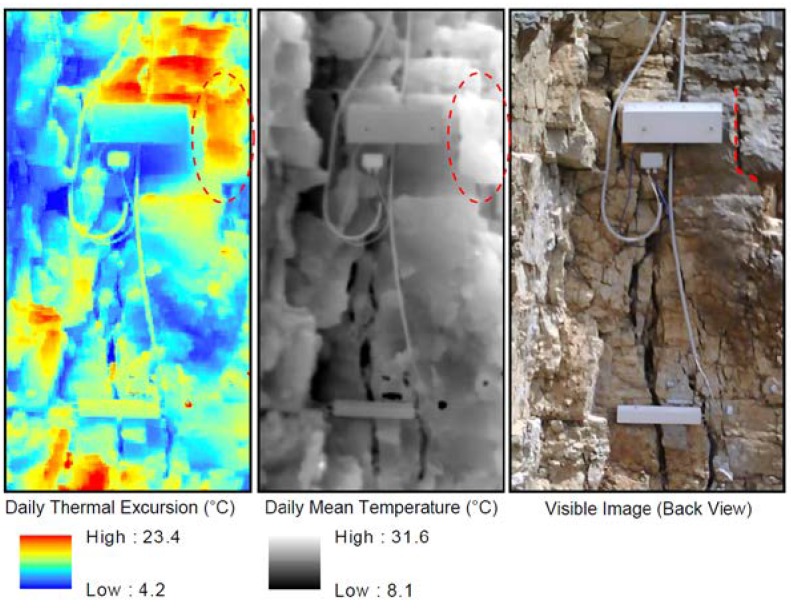
Example of 2D-analysis on temperature distribution derived by IR-Thermography. Spatial distribution of daily thermal excursion is here shown (left), highlighting the maximum daily excursion in correspondence of edges of prismatic rock-blocks isolated by conjugate joint sets. The analysis of daily mean temperature (centre) reveal a maximum located on a protruded irregular block (red dashed ellipse) isolated respect the rock wall behind by a vertical opened joint (dashed red line on right figure) across which a sharp contrast of daily thermal excursion and mean temperatures exists, confirming the role of joints in the amplitude of heating and cooling of rock mass within the thermal active layer.

The IR-images processed in a test-sector of the back face, highlights how the pixels which undergo maximum daily excursion are confined to small areas located in correspondence of edges of prismatic rock-blocks isolated by conjugate joint sets (Figure 13). For all the monitored seasons the daily mean temperatures reveal a maximum involving a protruded decametric-scale irregular block (red dashed ellipse in centre of Figure 13) isolated with respect to the rock wall behind by a vertical opened joint (dashed red line on Visible Image of Figure 13). This block, located in the most external part of the rock within the thermal active layer (Figure 1), also shows a marked daily thermal excursion with a sharp contrast with the rock behind, confirming the role of joints in the 2D-heat propagation. Across this opened interface, the amplitude of heating and cooling cycles decays acting as an impermeable thermal discontinuity (Figure 13), causing a differential heating of the block closer to the heat source.

No significant contrasts were observed, instead, in the other two main monitored joints as reported in Figure 4 (Joint 1 and Joint 2), because of their position at depth greater than the typical penetration depth, that is, at a distance from the front face greater than ones influenced by daily solar radiation.

**Figure 14:**
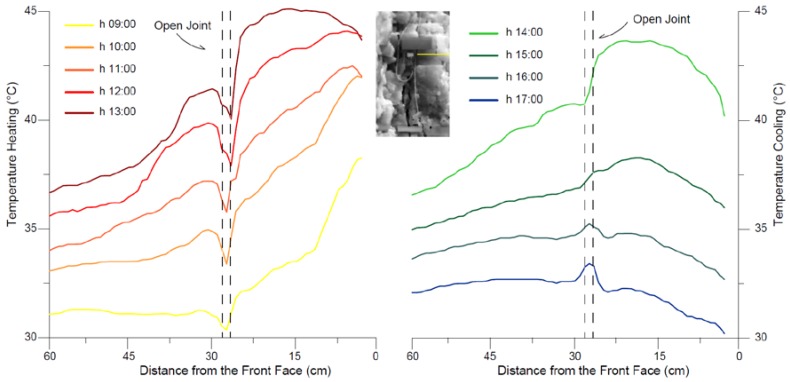
Temperature profiles derived across the protruded block of Figure 14 (yellow section) extract by hourly IR-image in both heating (**left**) and cooling (**right**) phases, as function of the distance from the exposed front face. Enhanced temperature contrasts across the bounding open joint (distance of 28 cm) were observed as effect of the breakage of thermal front propagation.

The intense differential heating across the most external joints (Figure 13) is detectable by the analysis of temperature profiles acquired from IR-images at different time of the day along a section involving the protruded irregular block (Figure 14).

At 9:00, when the solar radiation is perpendicular to the front face, the temperature begins to rapidly increase in the most external part of the rock cliff as direct effect of the direct radiation affecting the front face. During the heating phase the heat flux progressively propagates inside the rock mass in the semi-infinite space according to Carslaw and Jaeger equation [59], warming the rock mass.

Outside the TPD (i.e., greater distance from the front face), the temperature rise immediately decreases with an exponential function of the distance. Likewise, during the cooling phase (starts at 14:00) the temperature begins to decrease starting from the exposed front face, while, in depth, rock mass cool slower with a decay proportional to the magnitude applied thermal input and its position respect the heat source (TPD of Figure 3). A sharper decay is observed in presence of the open joint placed at distance of 28 cm from the exposed surface (Figure 13), where it maintains lower and higher temperatures respect the surrounding rock in heating and cooling phases respectively (Figure 14). Because of its opening, the joint is recognizable in the thermal profile due to the air filling the discontinuity (as already seen in Figure 10).

The presence of the open joint causes the fragmentation of the heat front propagation and a different amplitude of thermal cycles between the two portions of the rock mass, with temperature contrasts enhanced by the presence of joint in both heating and cooling phase (Figure 14). At the peak of heating phase at 12:00 a temperature contrast of about 7 °C across the open joint was experienced (Figure 14).

## 5. Discussions

The IRT survey technique was here applied to deduce a thermal response of a double exposed rock-block to solar radiation in daily and seasonal cycles, adopting 1D and 2D analysis. The thermal conductivity obtained by laboratory tests, allowed to define the heat propagation through a 1D conductive model, deriving information on thermal gradients in the rock matrix through the comparison among IRT-based temperatures (providing the surficial temperature distribution) and data from the rock thermometer (that monitors the rock temperature at depth of 8 cm). These parameters also allow to validate the temperature distribution resulting from IRT technique, comparing the latter with the theoretical temperature values deconvolved from depth to surface applying the Carslaw and Jaeger law (Figure 1, Figure 3 and Figure 8), thus demonstrating, for all seasons, the applicability of the IRT thermal survey to rock mass thermal response at different dimensional scales.

The IRT monitoring results allow a quantitative analysis on thermal rate of rock surface for both the back face (heating and cooling rates) and the front face (only cooling rate) of the monitored rock-block was carried out allowing to detect seasonal difference in the heat exchange velocity between environmental air and rock mass at its interface. This rate appears to be mainly influenced by intensity of direct solar radiation, by the surrounding environmental air temperature conditions, and, lastly, by the timing of the thermal maximum (function of the exposure of the rock-block). For this reason, both heating and cooling rates are higher in Autumn when the difference between the air temperature and the rock mass temperature is higher and heating and cooling of the rock mass are faster. As reported by other authors [14], thermal rates of rock mass can be strongly conditioned by the jointing conditions. In particular, it results that the higher is the rock jointing, the faster is the cooling rate.

Daily mean temperatures with relative spatial variability, under 2D conditions, were calculated for the four seasons, evidencing a direct correlation with the same seasons (i.e., maximum in Summer and minimum in Winter). Daily thermal excursion derived by IRT, instead, underlined the importance of solar radiation in the thermalisation of the rock surface, showing a maximum excursion in Autumn with respect to the other seasons, when moderately high solar radiation but low air temperatures coexist. On the contrary, the temperature recorded by rock thermometer in depth appears to be primarily related to air temperature, with smaller disturbances deriving by direct solar radiation.

The seasonal trend of the rock temperature at the rock surface was derived by averaging the monthly values referred to different seasons. For these reasons, the IRT technique is also suitable to study the long-period thermal signal that involves the entire monitoring period, evidencing the relations between surficial rock temperature and air temperature for both rock faces at different time-intervals. The long-term monitoring proved for the Winter season the occurrence of thermal inversion with the air, already starting from the daytime (14:00 for the front-face and from 17:00 for the back face). The thermal inversion is ascribable to lower radiative power of the sun in Winter season with respect the air temperature and to the exposure of the block. Possible night-time thermal inversions during the thermal monitoring carried out were not here analysed.

The IRT monitoring performed showed an accurate spatial and time resolution for the description of transient thermal input over the different seasons (Figure 7), as well as for the analysis of role of joints in the surficial heating of rock mass (Figure 10, Figure 13 and Figure 14). The analysis of thermal conditions of the rock-block across the main joints outcropping on the back face (Figure 10), despite its own exposure to the sunlight, highlighted the sensible decay of temperature inside the joints due to the different thermal diffusivity of the two media.

The observation of the temperature distributions resulting by multi-temporal 2D analysis (Figure 5) allows to evidence zones of the rock mass subjected to the most intense thermal forcing, as well as to characterise areas able to experience the maximum daily/annual thermal excursions, indicating sectors potentially predisposed to thermo-mechanical induced strain up to the onset of thermo-clastic processes (e.g., protruded block of Figure 13).

**Figure 15:**
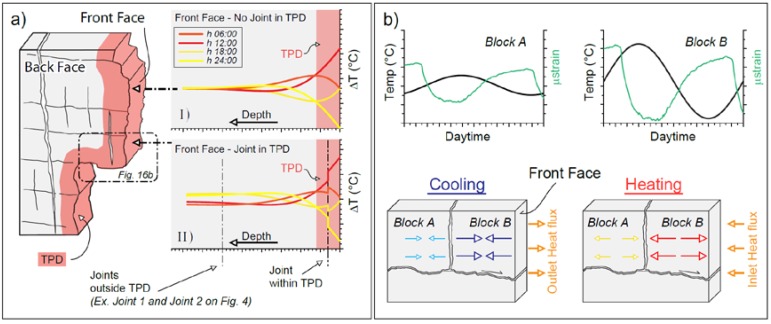
Conceptual model of heat transfer in a discontinuous rock mass on two different faces exposed to solar radiation and implications for thermo-mechanical induced strain. (**a**) Temperature profiles at different wave phases (qualitative coloured curves on Graph I, II) with respect to different jointing conditions on the exposed faces are shown. Temperature decays in depth and differential amplitude of daily thermal signal were appreciated only in the presence of open joints falling within the thermal active layer (Case II). (**b**) The thermal role of the joint can induce differential strain (green curves) in the two walls of the discontinuity (Block A and Block B) during a complete thermal cycle (heating and cooling).

The results of IRT monitoring performed on the selected rock-block adopting 1D and 2D analysis, allow to infer the role of persistent opened joints in the propagation of the heat front in depth, that is, inside the rock-block. Open joints falling within the thermal active layer can interrupt the propagation of the heat flux inside the rock, playing a strong role in the 3D-thermal behaviour of the jointed medium. This fragmentation of the thermal front which can results in a different thermalisation of the rock-block (Figure 12). In particular, where the solar radiation affects the Front face in absence of major joints, the temperature decays exponentially and undisturbed with depth (Figure 15, Graph I) according to the heat propagation law within continuous media (Equation (1)). The entity of this temperature reduction was derived by the comparison between the surficial rock interface and the rock thermometer (Figure 2 and Figure 4), which confirms the applicability of the IRT survey for the analysis of temperature distributions on large interfaces. Where open joints fall within the thermal active layer, instead, they can interact with the propagation of the heat flux from the front face towards the centre of the block, causing a fragmentation of the heat front and a thinning of the thermal active layer (Graph II on Figure 15). This effect is likely observed in Figure 13 and Figure 14, where sharp temperature contrast across the open joint exists. On the contrary, the open joint falling outside the typical depth of penetration of the heat front [22] (Figure 3) do not influence the heat propagation of the cyclical input, remaining outside the perturbed thermal layer (Figure 15, Graph II) as observed in joint 1 and 2 in Figure 10. The two open joints monitored by IRT on the back face, in fact, are located at depth greater than the TPD with respect to the frontal exposed face (Figure 2a and Figure 4), thus they do not provide an interference with the heat propagation.

In addition, because of this orientation respect to the back face (exposed itself to sunlight—Figure 6) and the large distance from the front face, no marked effect across the open joints were observed by IRT monitoring presented on Figure 10. On these joints, the heat flux propagates only in a direction normal to the back face and the temperature decays parallel to its direction, moving inside the rock mass and acting just as a non-conductive layer in the 3D heat propagation.

The differential heating of rock-blocks located within the thermal active layer (Graph II on Figure 15) and isolated by open joints from the rock wall behind (e.g., irregular block on Figure 13 and Figure 14), can results in a differential thermally-induced deformation, which is potentially able to drive, cumulatively on wide time scales, plastic deformation along joints, causing block instability. The protruded rock-blocks, if kinematically free to move and predisposed to planar failure, can be cyclically perturbed by periodic thermal input, which intensity decreases in depth as a function of the distance from the surface and the presence of thermal discontinuities (i.e., air interfaces).

Where open joints interact with the heat propagation within the most external portions of the rock mass, in fact, the most external block exposed to cyclical heating and cooling phases (Block B on Figure 15) can suffers larger thermal excursions respect to the Block A, located behind the opened joint (see graphs relative to block A and B on Figure 15). This effect is clearly observed in temperature profiles across the protruded rock-block reported on Figure 14. This varied heating across the open joint can reflects in differential cyclical thermo-mechanical induced strains (green curves on Figure 15), which inelastic contribution can contribute cumulatively to block instability.

The greatest thermal excursion at daily or seasonal time-windows (e.g., Autumn season), can be regarded as a possible mechanism responsible of thermo-mechanical inelastic strains along the joints and microcracks pervading the rock-block, which can cumulate over a long-period time window, by the repetitive accumulation of irreversible strains.

This thermally-driven mechanism, together with other elasto-plastic or viscous deformations, can represent a mechanism, even without the presence of a wedge-driving block [46,48].

## 6. Conclusions

Rock masses are exposed to daily and seasonal thermal cyclical inputs deriving by intense solar radiation whose action, repeated on wide time-windows, can be regarded as a preparatory action for slope deformations. In this frame, an exhaustive comprehension of the intensity and variability of thermal inputs acting on a rock mass is mandatory for the validation of cause-to-effect relation between thermal forcing and possible stress-strain response.

With this purpose, a multi-sensor thermal monitoring was carried out for two years across the four seasons by means of direct (i.e., type-K thermocouple) and passive InfraRed Thermographic (IRT) techniques. The multi-sensing monitoring allows to derive the amplitude of thermal input at the rock surface detailing the effect of solar paths and exposure in the thermalisation of a rock-block. Daily and seasonal monitoring of the rock-air interface allows to: (i) constrain the role of thermal radiation in the heating of a slope mass, (ii) evaluating the magnitude of heating and cooling rates as well as daily thermal excursion.

The obtained results confirmed the validity of the IRT technique, which was supported by analytic calculation through the deconvolution of the thermal signal derived by direct sensing considering a 1D heat propagation law in a semi-infinite space. The main outcomes also testify the suitability of the IRT to the characterisation of jointed rock masses with an appropriate time and space resolution: a combination of 1D and preliminar-2D analysis from IR-images was adopted, evidencing the suitability of the technique to the monitoring of jointed rock masses at large scale. The adopted analyses stressed the role of joints in heat propagation, highlighting possible implications for thermally-induced rock mass instabilities due to planar or wedge block failure mechanisms.

In addition, the IR-Thermography revealed to be a useful tool for the characterisation of the thermal effects on discontinuous rock mass along wide surfaces, whose understanding is fundamental and preparatory to the study of thermo-mechanical processes at rock-block scale, encouraging further applications of this technique in the studies of thermal weathering of natural rock aggregates and to the conservation of cultural heritage, as well as for investigating precursors of rockfalls.

These results represent reliable constraints for evaluating the surficial temperature distribution of jointed rock masses and suitable to validate numerical models devoted to establishing cause-effect relations between thermal stress and induced strains for assessing predisposing conditions for slope instability and mitigate the related risk.

## Figures and Tables

**Table 1:** Values of heating surficial rock temperature rate (HTRs), cooling surficial rock temperature rate (CTRs), heating thermal excursion (HTEx), cooling thermal excursion (CTEx) for the back side of the rock-block. The heating values were calculated taking into consideration a time interval ranging from 09:00 to 12:00 while the cooling values were calculated taking into account a time interval between 14:00 to 17:00.

Season	HTRs	CTRs	HTEx	CTEx
	°C/h	°C/h	°C	°C
Autumn	3.59	2.88	9.31	8.06
Winter	2.30	2.68	6.91	7.18
Spring	2.75	2.02	7.44	5.41
Summer	1.92	1.93	5.76	5.79

**Table 2:** Values of cooling surficial rock temperature rate (CTRs) and of cooling thermal excursion (CTEx) for the front face of the rock-block. The values were calculated taking into account a time interval from 12:00 to 15:00.

Season	HTRs	CTRs	HTEx	CTEx
	°C/h	°C/h	°C	°C
Autumn	n.a.	1.75	n.a.	5.26
Winter	n.a.	0.54	n.a.	1.61
Spring	n.a.	1.25	n.a.	3.75
Summer	n.a.	0.65	n.a.	1.95

**Table 3:** Spatial variability of Daily Mean Temperature and Thermal Excursion derived by 2D thermal image analysis.

Season	Daily Mean Temperature	Daily Thermal Excursion
	(°C)	(°C)
Spring	25.38 ± 1.97	10.88 ± 2.49
Summer	36.0 ± 1.56	11.81 ± 2.44
Autumn	20.77 ± 1.33	16.7 ± 2.92
Winter	11.34 ± 1.01	6.00 ± 1.13
